# State-multiplexing approach for optimized expansion of entanglement-based quantum networks

**DOI:** 10.1038/s41377-025-01892-0

**Published:** 2025-06-20

**Authors:** Anahita Khodadad Kashi, Michael Kues

**Affiliations:** 1 Institute of Photonics, Leibniz University Hannover, 30167 Hannover, Germany; 2 Cluster of Excellence PhoenixD (Photonics, Optics, Engineering – Innovation Across Disciplines), Leibniz University Hannover, 30167 Hannover, Germany

**Keywords:** Applied optics, Quantum optics

## Abstract

An optimized quantum network design is demonstrated by realizing a state-multiplexing quantum light source via a dual-excitation configuration technique. This approach optimizes the usage of the finite wavelength spectrum, facilitating the efficient expansion of entanglement-based fully-connected quantum networks across multiple users.

To date, quantum network designs and their implementations have undergone extensive exploration. Among different network topologies, fully-connected quantum network architectures^1–7^ – allowing each user to communicate simultaneously with any other user in the network by exclusively relying on point-to-point links – are favorable for large-scale entanglement-based quantum communications and distributed quantum computing spanning inter-continental distances. The fully-connected quantum network topology offers minimized congestion and need for buffering, offers maximal route redundancy, hence more resilience against link failures, and reduces the overall network costs. Moreover, by eliminating the need for trusted nodes, vulnerability to security loopholes in entanglement-based secure key establishment is minimized.

Probing resource-efficient schemes for scalable implementations of fully-connected quantum networks is essential for the future realization of the quantum internet. In recent years, by harnessing the advancements in wavelength division de/multiplexing components, fully-connected quantum networks have been realized^1,4,8,9^, benchmarking essential applications such as entanglement-based quantum key distribution protocols among multiple users. For an N-user quantum network, according to the graph theory, at least N×(N-1) links are required, which in experiments are realized through wavelength channels sharing quantum correlations. Due to the finite nature of the available wavelength spectrum in photon pair sources, the network expansion is inevitably constrained to a limited number of users^10–15^. This highlights the significance of probing solutions that could save on the available wavelength bandwidth. In some recent demonstrations, reconfigurable multiplexing techniques based on the wavelength selective switch technology have been utilized^5,7,16^ and a quadratic improvement in wavelength saving was achieved in a quantum network between 8 users using 16 wavelength channels^2^.

In a recent publication in Light: Science & Applications^17^, Yun-Ru Fan et al. report an optimized scheme for wavelength division multiplexing in entanglement-based quantum networks using a state-multiplexing quantum light source. In this approach, the researchers employ two lasers with different frequencies (the so-called dual pump configuration) to excite a third-order nonlinear component, i.e., a silicon-nitride microring resonator chip, facilitating simultaneous occurrence of one non-degenerate and two degenerate spontaneous four-wave mixing processes^18^. Using this technique, the emission wavelength overlap creates correlation between photons at a given wavelength with photons at three different wavelengths. Leveraging the state multiplexing scheme, in this approach, each user who occupies one wavelength resource can connect with the other three users. As an example (see Fig. 1), Charlie shares correlation through his frequency channel ω_2_ with other three users Bob, Ivan, and Alice, who are in possession of ω_-2_, ω_8_, and ω_18_, respectively. This enables Charlie to establish a simultaneous secure exchange of quantum information with these other three users. With this approach, the researchers have benchmarked the implementation of the entanglement-based BBM92 quantum key distribution protocol^19^ using time-energy entanglement in a fully-connected 4-user quantum network, using 6 wavelength channels only, thus saving on half of the available wavelengths compared with previous demonstrations^1^. In this implementation, the secret key rates among different users can be adjusted on-demand by balancing the generation efficiencies of the non-degenerate and degenerate SFWM processes^20–22^. Remarkably, in their research, the authors highlight the scalability potential of the state-multiplexing approach by showcasing a 10-user fully-connected quantum network requiring 34 rather than 90 wavelength channels, thus saving on 56 available wavelength channels.

**Fig. 1:**
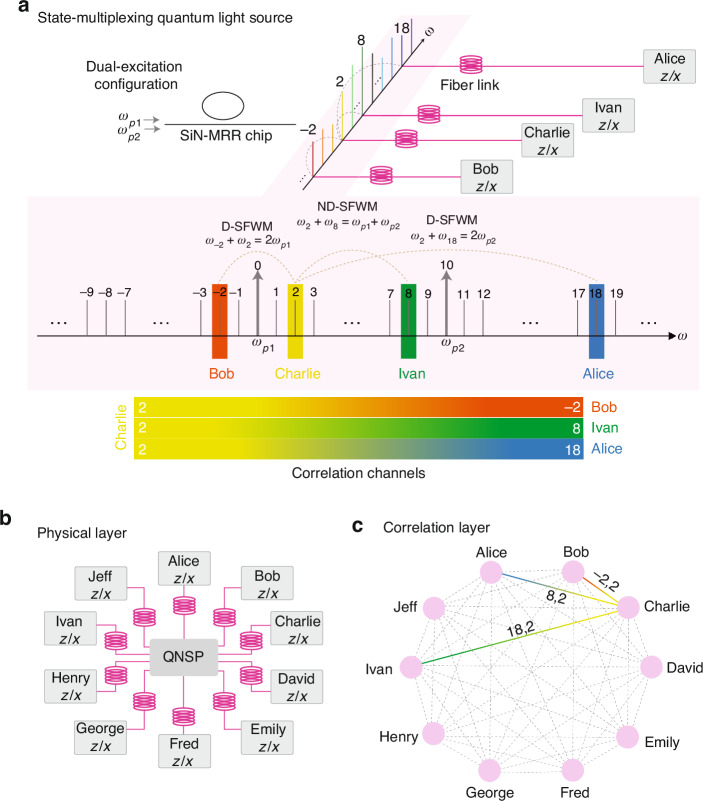
Conceptual demonstration of the state-multiplexing approach for the realization of a fully-connected quantum network. **a** Schematic illustration of the frequency correlations in the dual-excitation configuration scheme used for the state-multiplexing quantum light source. Each frequency channel is correlated with three different frequency channels through two degenerate (*ω*_2_ + *ω*_−2_ = 2*ω*_*p*1_, *ω*_2_ + *ω*_18_ = 2*ω*_*p*2_) and one non-degenerate (*ω*_2_ + *ω*_8_ = *ω*_*p*1_ + ω_*p*2_) SFWM process. **b** The physical layer of a 10-user fully-connected quantum network, where a centralized QNSP allocates a certain group of frequency channels to each user via frequency multiplexing components. The users’ hardware is composed of a balanced beam splitter, two unbalanced Michelson interferometers, and four single-photon detectors, enabling the users to perform random projection measurements in two mutually unbiased Z and X basis. **c** The correlation layer of a 10-user fully-connected quantum network. The correlation channels are shown with lines connecting the users. (SiN-MRR Silicon Nitride micro ring resonator, FSR free spectral range, D-SFWM degenerate spontaneous four-wave mixing, ND-SFWM non-degenerate spontaneous four-wave mixing, QNSP quantum network service provider)

The presented approach, i.e., the state-multiplexing quantum light source, has the potential to establish a scalable and reconfigurable fully-connected quantum network, paving the way for the future realization of the quantum internet. The state-multiplexing scheme can additionally be employed in quantum networks with various entanglement resources such as polarization and time-bin entanglement. With the growing size of the network, the pump configuration can be dynamically adjusted to address specific key exchange loads. For instance, a single wavelength could carry additional multiplexing states by employing three or more laser excitations, hence an enhanced scalability of the quantum network. Furthermore, a combined implementation of the state-multiplexing approach with time-sharing, beam splitter multiplexing^2^, and the flex grid technology^5–7^ allows for the transition towards meaningful large-scale implementations of fully-connected quantum networks.

In perspective, the state-multiplexing quantum light source combined with chip-integrated measurement basis analysis enables scalable implementation of quantum key distribution networks for the future realization of the quantum internet.
